# Dissecting the complexity of local and systemic circadian communication in plants

**DOI:** 10.1038/s44323-024-00003-3

**Published:** 2024-06-19

**Authors:** Mostafa Mortada, Lu Xiong, Paloma Mas

**Affiliations:** 1 Centre for Research in Agricultural Genomics (CRAG), CSIC-IRTA-UAB-UB, Campus UAB, Bellaterra 08193 Barcelona, Spain; 2 Consejo Superior de Investigaciones Científicas (CSIC), 08028 Barcelona, Spain

**Keywords:** Developmental biology, Plant sciences

## Abstract

The plant circadian clock regulates daily and seasonal rhythms of key biological processes, from growth and development to metabolism and physiology. Recent circadian research is moving beyond whole plants to specific cells, tissues, and organs. In this review, we summarize our understanding of circadian organization in plants, with a focus on communication and synchronization between circadian oscillators, also known as circadian coupling. We describe the different strengths of intercellular coupling and highlight recent advances supporting interorgan communication. Experimental and mathematical evidence suggests that plants precisely balance both the circadian autonomy of individual cellular clocks and synchronization between neighboring cells and across distal tissues and organs. This complex organization has probably evolved to optimize the specific functions of each cell type, tissue, or organ while sustaining global circadian coordination. Circadian coordination may be essential for proper regulation of growth, development, and responses to specific environmental conditions.

## Introduction

Many biological processes rhythmically oscillate in response to or in anticipation of the diel changes in environmental cues (primarily light and temperature). These 24-hour rhythms rely on robust and flexible internal cellular clocks^1,2^. For instance, circadian clocks are robust in their ability to maintain a nearly constant period within a physiological range of temperatures, thus defying the common influence of temperature on biochemical reactions. This is a remarkable property known as temperature compensation^3^. The circadian function is also flexible, as the circadian clock can re-entrain rhythms in response to unpredictable mismatched environmental cues. One familiar example of this flexibility is our capacity to recover from “jet-lag” when traveling across time zones. The recovery is due to the clock capability of eventually resynchronizing rhythms in phase with the new environmental conditions^4^. Circadian clocks also modulate or “gate” cellular responses to occur in a time-of-day specific manner^5^. Although circadian clocks are entrained by external cues^6^, rhythms are also sustained under constant conditions, indicating their endogenous nature^1^.

The circadian system was proposed to provide an adaptive advantage by enabling organisms to temporally coordinate key biological processes or clock outputs at the most favorable time-of-day^7^. The circadian activity is particularly relevant for plants. As sessile organisms, they need to accurately perceive and adjust to the changing environment, particularly during unfavorable environmental conditions^8,9^. The importance of circadian function in plants is manifested by the wide range of processes regulated by the clock: from growth and development to metabolism and responses to abiotic and biotic stresses. For instance, the circadian clock regulates seed germination, hypocotyl elongation, photosynthetic activity, mitochondrial function, the cell cycle, senescence, and responses to stress, to mention a few of many examples^10–14^. The generation of rhythms relies on timely synchronization by entraining cues such as light^15^. The intricate connection between light and the circadian clock also allows plants to precisely measure day-length as an indicator of changing seasons. This is particularly relevant in the control of seasonal processes such as the photoperiodic regulation of flowering time^16,17^.

The components and regulatory mechanisms responsible for the generation of rhythms have been extensively studied in the model system *Arabidopsis thaliana*. Core clock components regulate each other to define distinct peaks of expression and activity throughout the day and night^13,18,19^. The morning-expressed single-MYB domain transcription factors *CCA1* (*CIRCADIAN CLOCK ASSOCIATED 1*) and *LHY* (*LATE ELONGATED HYPOCOTYL*) are temporally followed by the sequential expression of members of the *PSEUDO-RESPONSE REGULATOR (PRR)* gene family (*PRR9*, *7*, 5 and *PRR1*/*TOC1*) from early morning to dusk. Other genes such as *ELF3* (*EARLY FLOWERING 3*), *ELF4*, and *LUX* (*LUX ARRHYTHMO*) are expressed at night^13,18,19^. Broadly speaking, the morning-expressed components repress genes expressed in the evening and vice versa. See comprehensive reviews^13,18^ for further details. In addition to transcription, proper timing of core clock expression and activity relies on changes in splicing, translational, and post-translational regulation as well as on rhythmic changes in chromatin marks^20–24^. Evidence of a non-transcriptional redox-based oscillator^25,26^ and circadian rhythms in Mg^2+^ involved in rhythmic ATP hydrolysis and translation rates^27^ have been proposed to represent ancient forms of conserved circadian metabolism^28^.

Recent research has expanded the study of plant circadian function beyond whole seedlings, focusing on specific cells, tissues, and organs. These studies have paved the way for analyses of local circadian coupling and long-distance communication. Excellent reviews have covered these topics, and readers are encouraged to consult them^29,30^. Here, we summarize current knowledge on the spatial organization of the plant circadian system, incorporating recent findings and revisiting the topic from a functional perspective. We also discuss the complexities of the circadian organization in plants, emphasizing the co-existence of autonomous yet interconnected clocks.

## Circadian coupling

Circadian coupling can be broadly defined as the capability of circadian oscillators to synchronize with each other. This synchronization, through intercellular or systemic communication, likely provides stability and circadian coordination among cells, tissues, and distant organs. The strength of intercellular coupling can vary, leading to diverse degrees of rhythmic coherence (synchronization) or autonomy (independence). Long-distance or systemic coupling largely refers to the interconnection and synchronization of rhythms across distinct tissues and organs. Experimental and mathematical evidence support the existence of autonomous, local, and distant circadian coupling. In the following sections, we briefly describe evidence supporting the different degrees of intercellular and systemic coupling.

### Weak intercellular circadian coupling in cotyledons and leaves

**Fig. 1:**
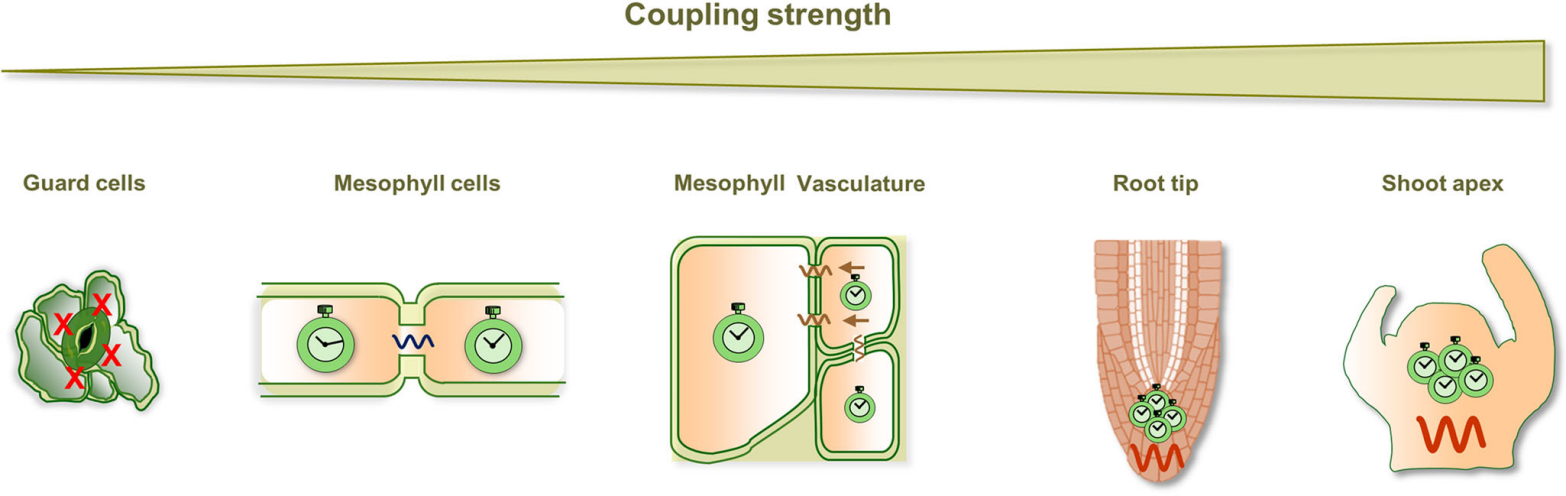
Varying degrees of intercellular coupling strength in plants. Schematic drawing depicting, from left to right, the increasing coupling strength in different cellular types. The lack of coupling of guard cells (represented by the red crosses) and the weak coupling between mesophyll cells (blue oscillatory waveform) are illustrated on the left. The vasculature showing intermediate coupling is represented in the middle (brownish oscillatory waveform), while the root tip and the shoot apex showing stronger coupling (red oscillatory waveform) are depicted on the right. The cells are not proportionally scaled for clarity. Figure created in part with BioRender.com. Please consult the text for further details.

Early studies in which the two cotyledons were grown under opposite entrainment cycles suggested a lack of circadian coupling, as rhythms maintained different phases over days^31^. Examining longer timeframes and closer leaf regions revealed a weak coupling with interesting phase-wave propagation and spirals of gene expression throughout the leaf ^32,33^. Analyses of single mesophyll cells of *Lemna gibba* also showed phase dispersion, albeit with spatially correlated phases, suggesting weak coupling^34^. Further analyses in leaves found that rhythms resynchronized to light–dark cycles more strongly than the cellular clocks coupled to each other^34,35^, again supporting a weak coupling. Overall, all the results aligned well with mathematical analyses^32,36,37^ and with studies showing heterogeneous circadian waveforms in excised leaves^36^. Thus, experimental and mathematical evidence support the notion of a weak intercellular coupling between proximal cellular clocks in leaves (Fig. 1).

Weak coupling in leaves does not pervade all cellular types. For example, guard cells were found to be out of synchrony with their neighboring cells, possibly indicating a lack of coupling^38^ (Fig. 1). Interestingly, guard cells lose plasmodesmata over development^39^; therefore, the loss of plasmodesmata could explain the lack of coupling, as plasmodesmata are likely a major route for circadian communication. As previously pointed out^29^, stochastic analyses predicted that noisy oscillators can desynchronize when weakly coupled^40^. This opens up the possibility of guard cell weak coupling. Another interesting example is the leaf vasculature, which displayed robust rhythms compared with neighboring mesophyll cells, suggesting a potentially strong coupling^32,36,41^ (Fig. 1).

### Strong intercellular circadian coupling in the shoot apex and in the root tip

Rhythms of detached shoot apexes showed homogeneous circadian waveforms sustained for an extended time^36^. Synchrony requires high cell density, potentially favoring intercellular coupling^36^ (Fig. 1). Mathematical modeling based on single-cell fluorescent reporter data indeed confirmed the strong coupling between shoot apex clock cells. Similar analyses also revealed weak coupling between mesophyll cells and intermediate coupling strength in the vasculature^36^. The results were consistent with the previous studies on leaves and on vascular systems, as mentioned above^32,41^. Strong coupling among the shoot apex clocks may confer improved resilience against genetic mutations and environmental perturbations^36^. Interestingly, in mammals, the strong intercellular coupling among neurons at the suprachiasmatic nucleus (SCN) counteracts the effects of mutations of key clock components^42,43^.

Strong circadian intercellular coupling is not limited to the shoot apex. For example, single-cell confocal imaging identified robust synchrony and cell-to-cell coupling at the root tip^44^ (Fig. 1). The analyses showed organ-specific circadian phases and waves of clock gene expression both within and between organs^44^. Mathematical models suggested faster-oscillating clocks in the shoot and root tip compared with other root regions, whereas separate organs displayed different circadian phases^45,46^, which agrees with prior observations^36,47^. Intercellular coupling rather than long-distance communication was proposed to drive the spatial waves of gene expression^45,46^. However, additional studies have proposed that continuous phase resetting at the root tip, and not cellular coupling, was responsible for wave formation^48^. Root elongation also contributes to the generation of spatial patterns of circadian expression^48^. Further studies are required to identify all the factors underlying these spatial waves of gene expression.

Strong coupling is found in areas of high cell density, like the root tip and shoot apex^36,44^. A recent study showed that while isolated leaf-derived cells maintained rhythms that responded to light and temperature cues, rhythmic stability improved with higher cell density^49^. The circadian clock association with both the endocycle and mitotic cycle^50^ opens the possibility of differential coupling strength between actively dividing and other cells. Consistently, local intercellular coupling was found to synchronize circadian rhythms in *Lemna* proliferating cells, driving the circadian waves of clock gene expression during frond proliferation^51^.

### Long-distance circadian communication

Shoots and roots communicate by exchanging photoassimilates, water, and nutrients. In addition to its role in controlling the timing of this systemic communication, the circadian clock uses the plant transport system to coordinate rhythms among distant parts of the plant (see below). Early studies showing tissue-specific rhythmic differences and self-sustained rhythms in cells, tissues, and organs^29,30^ argue against such distal coordination. However, shoot-root communication is important for regulating oscillator gene expression, rhythmic accuracy, and growth (see below). This could be an analogous situation to that described in mammals, whereby peripheral clocks sustain rhythms autonomously^52^ but long-distance signals coordinated from the SCN are crucial for overall circadian coordination^53,54^.

**Fig. 2:**
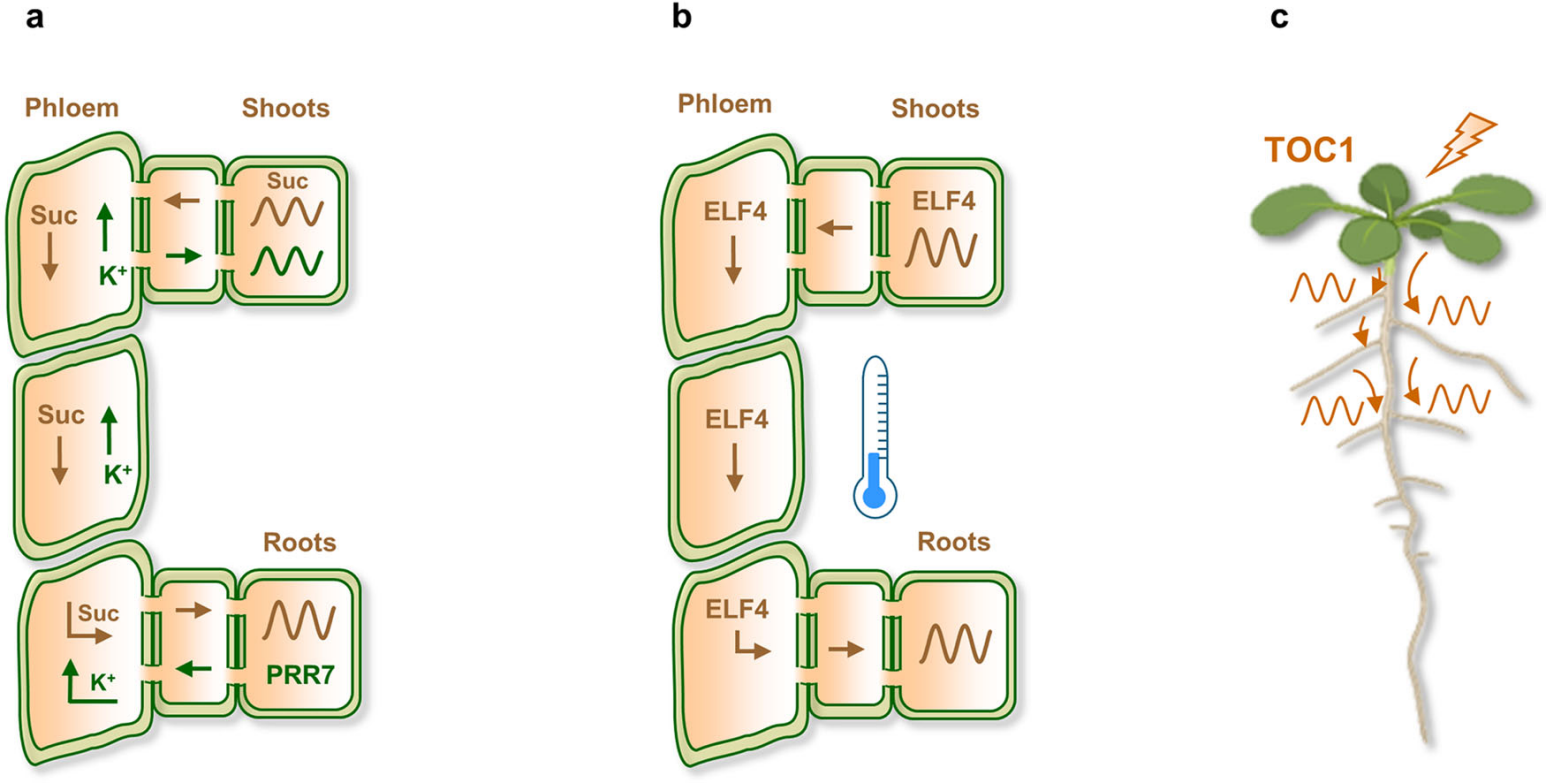
Long-distance shoot and root circadian communication is important for oscillator gene expression, rhythmic accuracy, and growth. **a** A synchronizing photosynthetic-derived signal (probably sucrose, suc) from shoots is important to set the rhythms in roots. The long-distance circadian communication is bidirectional. The clock component PRR7 regulates cation transport (K^+^) from roots into shoots, resulting in improved precision of rhythms in shoots. **b** The clock component ELF4 moves from shoots to influence rhythms in roots primarily at low temperatures (indicated by the blue thermometer). **c** Light applied only to aerial parts is sufficient for root hair growth rhythmicity, in a process that requires the function of TOC1 in shoots. Communication from shoots to roots is depicted by the brownish color while the green color represents the root-to-shoot communication. Figure created in part with BioRender.com. Please consult the text for further details.

Studies with detached organs supplemented with sucrose and cell-type specific analyses have shown autonomous rhythms in roots^36,44,45,55–58^. However, despite this root circadian autonomy, the shoot clock also influences rhythms in roots. For instance, an early study showed a synchronizing signal between shoots and roots that depended on photosynthetic activity^56^. This suggests the importance of daily fluctuations in carbohydrate flow from shoots to roots^56^ (Fig. 2a). The results are consistent with subsequent studies showing dampened rhythms in detached roots growing without sucrose^36^ and with the importance of sucrose signaling regulating the clock^59–61^. Light piping through the roots affects the circadian period, which establishes another route for circadian coordination^62^. Overall, the results suggest that organ-specific rhythms coexist with long-distance shoot-to-root circadian communication.

Laser microdissection to excise the shoot apex and micrografting experiments using WT and clock mutants also revealed the influence of shoots on rhythms in roots^36^. Alteration of plasmodesmata, a major route for circadian communication, also affected root rhythms. Interestingly, the circadian clock gates light-induced plasmodesmata transport during the day^63^. Regarding the signaling factors, experimental and mathematical evidence showed that the clock component ELF4 moves from shoots to regulate rhythms in roots^58^. Low temperatures favor ELF4 mobility, resulting in a slow-paced root clock, while high temperatures decrease movement, leading to a faster clock^58^ (Fig. 2b). The results also opened the possibility of two differentiated pools of ELF4 protein (mobile and static) with different functionalities.

A recent study has intriguingly shown bioluminescence rhythms generated at the organismal level^64^. The authors transfected duckweed (*Lemna minor*) cells with a dual reporter system to monitor both the well-established Arabidopsis clock gene *CCA1* and the Cauliflower mosaic virus 35S (CaMV35S) promoter. Notably, both reporters displayed bioluminescence rhythms, but with unique properties: while disruption of the circadian function significantly altered *CCA1* rhythmic expression, the CaMV35S rhythms remained unaffected. Instead, plasmolysis abolished the CaMV35S rhythms, suggesting a symplast/apoplast-mediated circadian rhythm generated at the organismal level, and independent of the canonical circadian clock. The results align well with both cell-autonomous and non-cell-autonomous rhythms even without regulation by the canonical circadian gene circuit^64^. In addition, the application of a single external entrainment cue to nearly fully desynchronized plants simultaneously elicited phase-dependent responses within the whole plant and with organ-specific properties^65^. The observed differences might be due to variations in circadian coupling in the different organs. Additionally, organ-specific variations in the sensitivities to the input cues and/or functional changes in the oscillator regulatory network may also contribute to the phase-dependent differences.

Long-distance communication modulates not only the rhythms of oscillator gene expression but also clock outputs such as growth and development. For instance, a recent study showed that root hair elongation is controlled by the shoot circadian clock^66^ (Fig. 2c). Indeed, light applied only to the aerial parts is sufficient for root hair rhythmicity^66^. Grafting experiments also showed that the circadian clock component TOC1 from shoots contributes to the generation of root hair rhythms^66^. Therefore, the study reveals the dominance of shoots over roots and the importance of systemic signaling for rhythmic root hair growth. Notably, long-distance circadian communication is bidirectional, as xylem sap analyses and grafting assays showed that the clock component PRR7 regulates cation transport from roots to shoots, resulting in improved rhythmic precision in shoots^67^ (Fig. 2a). In mammals, bidirectional circadian coordination has also been reported between the SCN and peripheral clocks^68^.

### Plant circadian organization: biological relevance

The different degrees of coupling strength and the coordination of rhythms along distant parts of the plant suggest a hierarchical structure of the plant circadian system. However, as described above, circadian organization in plants seems quite complex. The circadian system may have evolved to optimize the functional specificity of each cell type, tissue, or organ while still sustaining a degree of global circadian coordination across the whole plant. This coordination may be essential for the proper regulation of growth, development, and in response to specific environmental conditions. Thus, both cell-type specific autonomy and whole-plant coordination coexist within the plant circadian system.

The circadian particularities of the shoot apex and root tip initially suggested a hierarchical structure of the clock^29,30^. Strong coupling may facilitate a synchronized response to external changes, including abiotic or biotic stress, which is particularly important for cells that are buried and shielded from the environment. The distinctive waveforms observed in jet-lag experiments further suggest the potentially high sensitivity of shoot apex clocks to environmental changes^36^. Meristem cells in shoots and roots respond differently to DNA damage compared with differentiating cells^69^. Timely communication between cells may be crucial for rapid responses when genome integrity is compromised. Further, the link of the circadian function with the mitotic cycle and the endocycle^50,70^ opens the possibility that intercellular coupling may also aid in the precise temporal coordination of cell division and growth. Overall, strong coupling may be essential for orchestrating responses to environmental cues, managing stress, maintaining genome integrity, and coordinating cell division and growth.

The weaker coupling between leaf cells likely provides flexible responses to the rhythmic clock. As leaves are generally exposed to environmental light conditions, it is possible that strong coupling is not necessary for synchronization. This agrees with the finding that rhythms in leaves resynchronized more strongly to light–dark cues than via coupling^35^ and with the desynchronization of *Lemna gibba* cells in the absence of entrainment cues^34^. An analogous situation has been described in animal peripheral clocks showing coherent rhythms under entrainment that rapidly desynchronized when external synchronizing cues were removed^43^. Furthermore, the diurnal fluctuations in sugar production in leaves likely shape the circadian system differently from other non-photosynthetic cell types. Interestingly, the local coupling can minimize time errors under noisy light–dark cycles while maintaining organ-specific circadian phases^46^. It is worth noting that the coupling strength between leaf cells may vary under stress conditions, when a more coordinated response may be important for survival.

The specificity of rhythms in the plant vasculature^41^ opens the possibility that the vascular system may be a regulatory checkpoint for long-distance circadian communication. This could facilitate the movement of signaling molecules at a specific time-of-day or under particular conditions, influencing rhythmic processes in different organs. As mentioned above, ELF4 moves from shoot-to-root through the vasculature to modulate rhythms in a temperature-dependent manner^58^. Photoassimilates represent another interesting example. A balanced source-to-sink flow is essential for maintaining energy resources leading to a regulated balance for timely growth. Integrative mathematical analyses also suggest that long-distance communication and light sensitivity may also contribute to the observed period differences between organs^46^. Recent findings of bidirectional communication through root-to-shoot transport of water and nutrients^67^ pave the way for exploring systemic signaling in the control of specific clock outputs in plants.

## Perspectives

Interpreting plant circadian communication is challenging due to the complex structure of both plants and the circadian system. Factors like growth, development, and physiology influence protein exchange between cells^71^, most likely affecting the coupling strength. Young and senescent leaves show differences in circadian period^72^ while the coupling is likely connected with cell division, as mentioned above. In addition, local and long-distance communication may vary depending on stress, growth conditions (light, temperature, humidity), and growth medium or soil composition. For example, cell-autonomous circadian oscillation in roots appeared to be increased by sucrose in the medium^36,48^. This complexity can lead to seemingly contradictory results regarding local and systemic communication when plants growing under different conditions or at different developmental stages are examined.

**Fig. 3:**
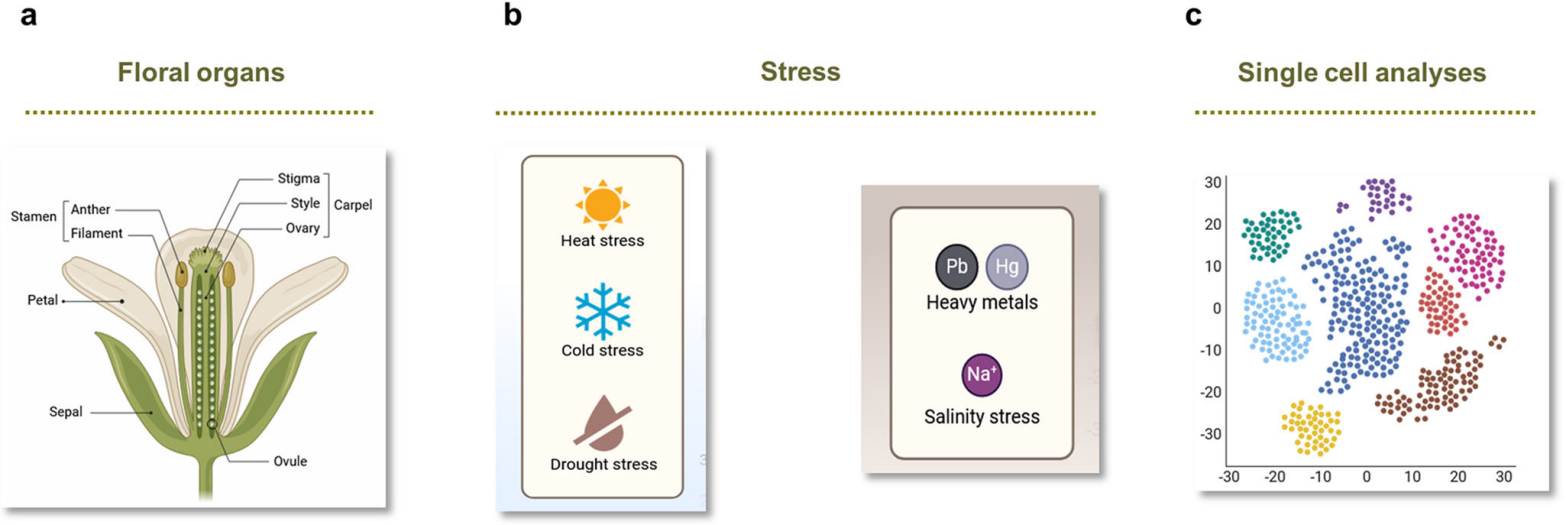
Prospective studies on local and systemic circadian communication in plants. Schematic drawing depicting some tentative future areas of research on circadian communication. **a** Analyses of local coupling between cells of the different floral organs as well as studies on the systemic communication of flowers with the rest of the plant. **b** Assessment of the influence of different stresses (heat, cold, drought, etc) and growth medium or soil composition on local and systemic coupling. **c** Single-cell RNA sequencing (scRNA-seq) analyses to obtain insights into cell synchrony and cellular coupling. Figure created in part with BioRender.com. Please consult the text for further details.

Further studies should examine the coupling strength in other tissues or cell types. For instance, detached pistils, the female reproductive organs, show highly synchronized and robust rhythms over extended time^73^. Although the circadian coupling was not formally studied, the pistil rhythms resembled those observed in organs with strong intercellular coupling. Therefore, it would be interesting to identify whether the clocks in pistils are coupled and, if so, to understand the biological relevance of such coupling (Fig. 3). This topic is of interest based on the importance of the circadian clock controlling many aspects of flower organ growth, development, and function^73–78^. Understanding the intricacies of circadian intercellular coupling in reproductive organs may be useful for optimizing reproduction and productivity.

Single-cell RNA sequencing (scRNA-seq) offers a promising yet technically challenging avenue for future research^79^. Initial circadian studies using scRNA-seq^70,80,81^ are paving the way for more detailed analyses of cell synchrony. A recent study combined scRNA-seq with bulk RNA-seq time series of leaves to estimate cell-type-specific gene expression^81^. By analyzing shifts in cell-type proportions over a diurnal time series they found that epidermal expression is maximized in the morning, while vascular expression peaks in the afternoon. Although the authors used a dataset from plants grown under continuous light (which disrupts the clock), the authors argue that the individual cells were still robustly rhythmic^81^. Phloem parenchyma expression peaked at night^81^, which aligns with the transitioning time, from sugar loading via plasmodesmata to active apoplastic movement^82^. Ideally, future scRNA-seq studies should integrate temporal and spatial resolution to create a comprehensive view of circadian regulation within different cell types (Fig. 3). These analyses could also uncover cell-specific sensitivities to input cues. For instance, water and nutrients may be stronger entrainment cues in roots than in shoots^55,67^. Furthermore, exploring cell-type specific associations with specific outputs, like the role of vascular phloem companion cells in flowering time regulation^41^, could yield valuable insights into the spatiotemporal intricacies of clock function.

Cell-type specific variations in the oscillator regulatory network may explain differences in how cells respond to input signals and adjust clock outputs^45,83^. Previous studies have already shown clock proteins with organ-specific regulatory functions^84,85^. However, more comprehensive and integrative analyses are required. For example, a recent study combined light sensitivity with local and long-distance circadian communication into a clock model^46^. Integrating cell-type specific gene expression patterns with specific inputs and outputs regulated by the spatial control of oscillator components is a complex challenge. Initial steps have been taken, and further research is likely to provide a deeper understanding of the spatiotemporal function of the plant circadian system.
